# *Methanonatronarchaeia* are deep-branching ancestrally methanogenic archaea distant from *Halobacteria*

**DOI:** 10.1093/ismeco/ycag071

**Published:** 2026-03-23

**Authors:** Brittany A Baker, Romain B Leroy, Purificación López-García, Laura Eme, David Moreira

**Affiliations:** Unit Evolutionary Biology of the Microbial Cell, Department of Microbiology, Institut Pasteur, 75015 Paris, France; Ecologie Société Evolution, Université Paris-Saclay, AgroParisTech, CNRS, 91190 Gif-sur-Yvette, France; Ecologie Société Evolution, Université Paris-Saclay, AgroParisTech, CNRS, 91190 Gif-sur-Yvette, France; Ecologie Société Evolution, Université Paris-Saclay, AgroParisTech, CNRS, 91190 Gif-sur-Yvette, France; Department of Cell and Molecular Biology, University of Rhode Island, Kingston, RI 02881, United States; Ecologie Société Evolution, Université Paris-Saclay, AgroParisTech, CNRS, 91190 Gif-sur-Yvette, France

**Keywords:** halophilic archaea, phylogenomics, methanogenesis, compositional bias

## Abstract

Since their discovery, the phylogenetic placement of the extremely halophilic, methanogenic *Methanonatronarchaeia* has remained controversial. Different studies have variably placed this lineage as sister to the archaeal class *Halobacteria* (haloarchaea) or as a deep-branching euryarchaeal group. These conflicting results may reflect methodological artefacts linked to the strong amino acid compositional bias characteristic of halophilic archaea and evolutionary model misspecification. Here, we reanalyse published phylogenomic datasets using site-heterogeneous mixture models that mitigate such biases. Our analyses consistently recover *Methanonatronarchaeia* as a deep-branching lineage basal to the *Methanotecta*, independent of the inclusion of the recently described *Ordosarchaeia*. We further show that *Ordosarchaeia* do not constitute a distinct lineage but fall within the previously described *Halorutilales* and *Afararchaeaceae*. Re-examination of the methyl-coenzyme M reductase phylogeny indicates that the placement of *Methanonatronarchaeia mcr* genes is best explained by vertical inheritance, without invoking horizontal gene transfer from unknown donors. Together, our results support ancestral methanogenesis within this lineage and its independent adaptation to extreme halophily.

## Introduction

Since their description in 2017, the phylogenetic placement of the class *Methanonatronarchaeia*—an extremely halophilic, methyl-reducing methanogenic lineage composed of the orders *Methanonatronarchaeales* and JAANXF01—has remained debated [1–5]. Early phylogenetic analyses based on ribosomal proteins placed them as the closest sister lineage to the class *Halobacteria* [4]. However, subsequent studies incorporating broader taxon sampling, alternative phylogenetic markers, and more sophisticated sequence evolution models [1–3] instead recovered *Methanonatronarchaeia* as a deeper-branching euryarchaeal lineage, placed at the base of the *Methanotecta* (i.e. the group encompassing *Halobacteria, Archaeoglobales, Methanophagales* (ANME-1), *Syntrophoarchaeales, Methanosarcinales, Methanomicrobiales*, and *Methanocellales*). Extremely halophilic archaeal lineages (including *Halobacteria, Methanonatronarchaeia, Nanohaloarchaeota*, and *Halarchaeoplasmatales*) have evolved remarkable adaptations to counterbalance the osmotic stress imposed by their hypersaline habitats. They actively accumulate molar concentrations of potassium in their cytoplasm [6] and, to maintain protein conformation and activity under such near-saturating salt concentrations, their proteomes are highly acidic, enriched in aspartic and glutamic acids, and depleted in basic and large hydrophobic amino acids [7–9]. This amino acid usage bias, combined with the high evolutionary rate of some lineages like the *Halobacteriales*, can lead to phylogenetic reconstruction artefacts, such as long-branch attraction, which can explain the conflicting placements of *Methanonatronarchaeia*.

Phylogenetic analyses can also be strongly affected, often positively, by the incorporation of new taxa, especially when they break long branches [10], such as the basal one of *Halobacteria*. Recently, Zhao *et al*. described the *Ordosarchaeia*, a new hyperhalophilic archaeal lineage from soda–saline lakes [11]. A phylogenetic analysis based on 53 conserved archaeal proteins [12] robustly placed them as the sister group to *Halobacteria* (with 100% ultrafast bootstrap, UFB) and, most interestingly, *Methanonatronarchaeia* branched sister to the *Ordosarchaeia* + *Halobacteria* clade (90% UFB). By contrast, excluding *Ordosarchaeia* placed *Methanonatronarchaeia* in a much deeper position, far from *Halobacteria*, similar to previous studies [1–5]. Based on these results, the authors concluded that incorporating *Ordosarchaeia* into phylogenomic analyses ‘clarifies the sisterhood of *Methanonatronarchaeia* with *Halobacteria’*. In fact, this potential grouping of *Methanonatronarchaeia* and *Halobacteria* has important evolutionary implications, as it supports a single evolutionary origin of extreme halophily in an ancestor of both groups, in contrast with two independent origins in the case of a deeper phylogenetic position of *Methanonatronarchaeia* [1–5].

It also has implications for our understanding of the evolution of methanogenesis in archaea. Previous analyses have shown that *Halobacteria* are closely related to several groups of methanogenic archaea, in particular the *Methanomicrobiales*, and are therefore inferred to have evolved from a methanogenic ancestor that lost methanogenesis secondarily [1–5, 13]. Zhao *et al*. analysed the phylogeny of the different subunits of the key terminal enzyme of methanogenesis, the methyl-coenzyme M reductase (McrA, B, C, D, and G), and found that the *Methanonatronarchaeia* sequences are not closely related to those of *Methanomicrobiales*, which they interpret as evidence that *Methanonatronarchaeia* acquired the *mcr* genes secondarily through horizontal gene transfer (HGT) from an unknown, distant methanogenic donor [11].

## Results and discussion

**Figure 1:**
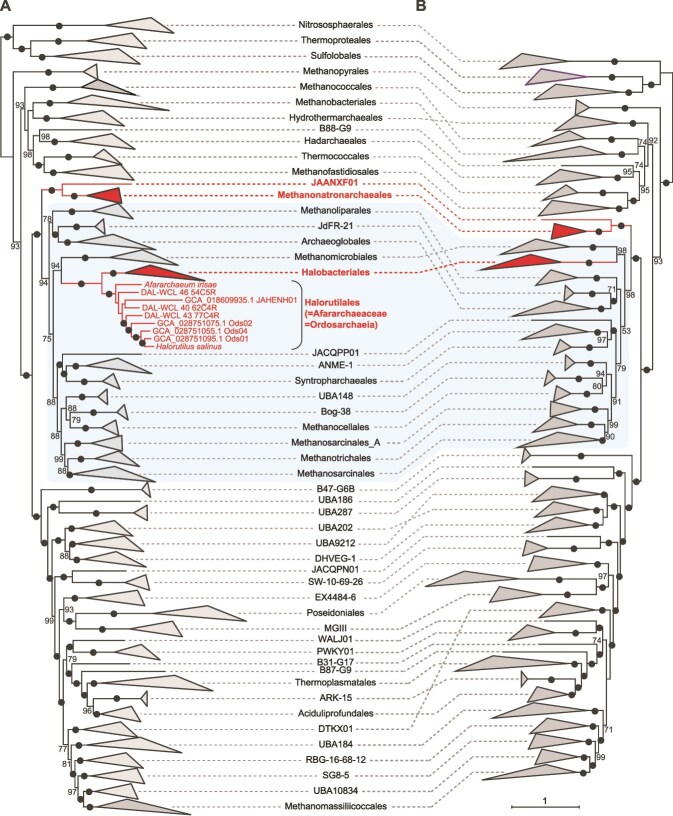
Maximum likelihood phylogenetic analysis of archaea; (A) tree containing 1655 taxa, including the newly described halophilic *Ordosarchaeia* and closely related taxa (*Afararchaeaceae* and *Halorutilales*); (B) tree containing 1646 taxa, excluding these new halophilic taxa; different archaeal groups are named according to the Genome Taxonomy Database (GTDB; https://gtdb.ecogenomic.org/) release r226; the trees were reconstructed with IQ-TREE (https://iqtree.github.io/) and the LG + C20 + G4 mixture model using 53 conserved markers (12 943 sites) and 1000 ultrafast bootstraps, and arbitrarily rooted on the *Nitrososphaerales* + *Thermoproteales* + *Sulfolobales* branch; numbers at branches are ultrafast bootstrap support values, with black dots representing 100% (only values >50% are shown); the superclass *Methanotecta* is highlighted with a background. Complete trees are shown in Supplementary Fig. S1.

A possible limitation of these conclusions is that they rely on phylogenetic trees inferred using simple, site-homogeneous models of sequence evolution (e.g. LG + F + G4). These models apply a single substitution matrix to all sites of the alignment and assume a single set of amino acid frequencies at equilibrium, so that different substitution preferences at different sites are not taken into account, which, in the presence of strong compositional biases as in halophilic archaea, can induce a systematic error in the form of a compositional attraction artefact [1–5, 14]. We therefore re-analysed Zhao *et al*.’s dataset [11] using a more realistic site-heterogeneous mixture model (LG + C20 + G4) [15]. We recovered *Methanonatronarchaeia* in a deep-branching position, basal to the *Methanotecta*, far from *Halobacteria* (Fig. 1A). Notably, this deeper-branching placement is robust to the inclusion or not of *Ordosarchaeia* (Fig. 1B). Furthermore, our phylogenetic analysis indicates that *Ordosarchaeia* do not define a new distinct lineage, but instead are nested within previously described halophilic archaea (Fig. 1A), including members of the order *Halorutilales* [16] and the family *Afararchaeaceae* [2]. *Halorutilales*, defined by the cultured species *Halorutilus salinus*, were the first to be described, giving this name taxonomic priority, and making *Afararchaeaceae* and *Ordosarchaeia* later synonyms (i.e. names published after the first valid name [17]).

**Figure 2:**
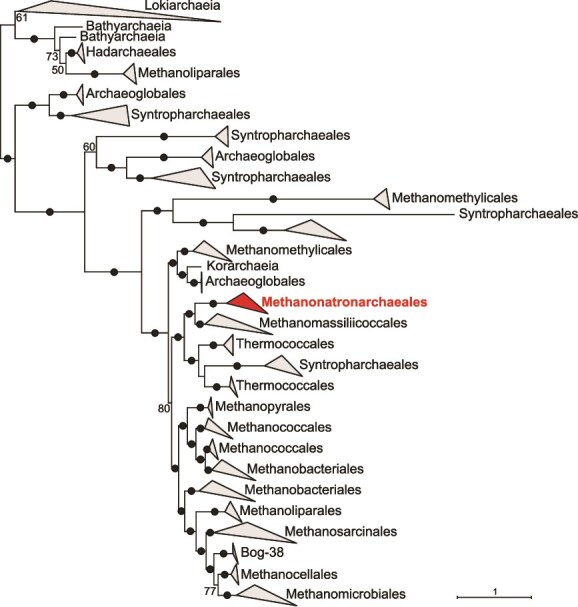
Maximum likelihood phylogenetic analysis of McrA; different archaeal groups are named according to the Genome Taxonomy Database (GTDB; https://gtdb.ecogenomic.org/) release r226; the tree was reconstructed with IQ-TREE (https://iqtree.github.io/) using 653 taxa and 630 sites with the LG + C20 + G4 mixture model and 1000 ultrafast bootstraps, and arbitrarily rooted on the *Lokiarchaeia* and closely related taxa; numbers at branches are ultrafast bootstrap support values, with black dots representing 100%; the complete tree is shown in Supplementary Fig. S2.

These phylogenomic results prompted us to re-examine the scenario proposed by Zhao *et al*. for the evolution of the *mcr* genes in *Methanonatronarchaeia*. We reconstructed the phylogeny of the essential catalytic McrA subunit using the LG + C20 + G4 mixture model. The resulting tree (Fig. 2), which does not perfectly mirror the corresponding species tree (Fig. 1A), shows that this enzyme has a very complex evolutionary history most likely involving multiple HGT events [18–22]. As in previous analyses, the methanonatronarchaeial McrA sequences branch close to those of *Methanomassiliicoccales*, suggesting HGT between the two groups, although the direction of transfer remains to be determined. They are, however, very far from the closest methanogenic relatives of *Halobacteriales*, namely the *Methanomicrobiales*. In light of the deep position of *Methanonatronarchaeia* in the species tree reconstructed using a mixture model (Fig. 1A), HGT does not need to be invoked to explain the deep phylogenetic placement of their McrA homologues. In fact, the position of *Methanonatronarchaeia* in the McrA tree is compatible with a scenario of general vertical inheritance of the *mcr* genes within the *Euryarchaeota* with subsequent loss of these genes along the branch leading to *Halorutilales* and *Halobacteriales*.

In conclusion, our reanalysis of the position of *Methanonatronarchaeia* indicates that the close relationship to *Halobacteria* found by Zhao *et al*. was most likely due to the inadequacy of the simple sequence evolution model used in their analysis. A more sophisticated mixture model supports a deeper position at the base of the *Methanotecta*, implying an independent adaptation of *Methanonatronarchaeia* to hyperhalophy and the vertical inheritance of the methanogenesis pathway *mcr* genes.

## Supplementary Material

ycag071_Baker_et_al_Supplementary_figures

## Data Availability

Multiple sequence alignments have been deposited in Figshare (https://figshare.com/s/bcf46d109a4655de5dc7).
