# Supplementary material for: Methanonatronarchaeia are deep-branching ancestrally methanogenic archaea distant from Halobacteria
Source: ISME Commun. 2026 Mar 23;6(1):ycag071. doi: 10.1093/ismeco/ycag071 (PMC13082222; doi:10.1093/ismeco/ycag071)
Supplement: ycag071_Baker_et_al_Supplementary_figures [file ycag071_baker_et_al_supplementary_figures.pdf]

A

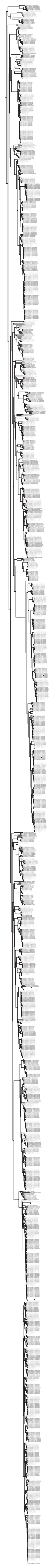

B

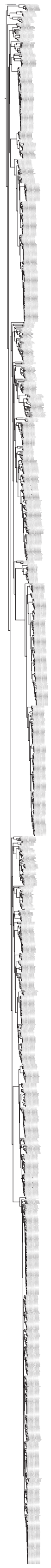

Figure S1. Maximum likelihood phylogenetic analysis of archaea. A) Tree containing 1,655 taxa, including the newly described halophilic Ordosarchaeia and closely related taxa (Afararchaeaceae and Halorutilales). B) Tree containing 1,646 taxa, excluding these new halophilic taxa. Different archaeal groups are named according to the Genome Taxonomy Database (GTDB) release (r226). The trees were reconstructed with IQ-TREE v. 2.0.3 and the LG+C20+G4 mixture model using 53 conserved markers (12,943 sites) and 1000 ultrafast bootstraps, and arbitrarily rooted on the Nitrososphaerales + Thermoproteales + Sulfolobales branch. Numbers at branches are ultrafast bootstrap support values.

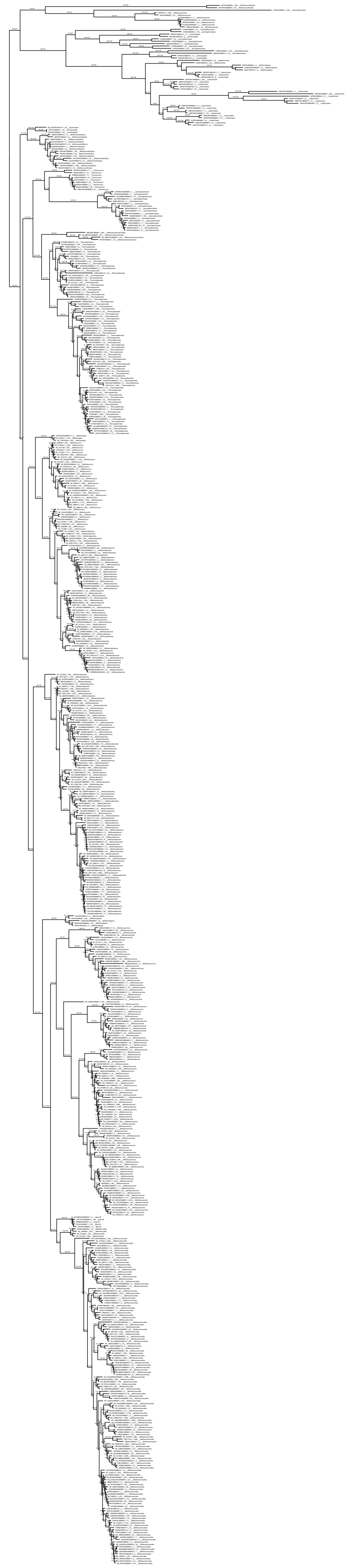

Figure S2. Maximum likelihood phylogenetic analysis of McrA. Different archaeal groups are named according to the Genome Taxonomy Database (GTDB) release (r226). The tree was reconstructed with IQ-TREE v. 3.0.1 using 653 taxa and 630 sites with the LG+C20+G4 mixture model and 1000 ultrafast bootstraps, and arbitrarily rooted on the Lokiarchaeia and closely related taxa. Numbers at branches are ultrafast bootstrap support values.
